# Colorectal cancer screening participation among citizens not recommended to be screened: a cohort study

**DOI:** 10.1186/s12876-022-02331-9

**Published:** 2022-05-20

**Authors:** Pernille Thordal Larsen, Susanne Fogh Jørgensen, Sisse Helle Njor

**Affiliations:** 1 University Research Clinic for Cancer Screening, Department of Public Health Programmes, Randers Regional Hospital, Skovlyvej 15, 8930 Randers NØ, Denmark; 2 Department of Clinical Medicine, Aarhus University, Aarhus, Denmark; 3 Danish Bowel Cancer Screening Database, Aarhus N, Denmark

**Keywords:** FIT screening, Screening organization, Participation, Prevention, Inflammatory bowel disease, High-risk individuals

## Abstract

**Background:**

Guidelines on colorectal cancer (CRC) screening recommend screening of average-risk adults only. In addition, screening of individuals with active inflammatory bowel disease (IBD) might result in too many false-positive cases. However, the organisers of CRC screening programmes are often uninformed of whom to exclude due to an elevated CRC risk or active IBD. It is therefore unknown how often high-risk individuals (i.e. individuals with a previous diagnosis of CRC or polyps associated with hereditary CRC syndromes and certain patient groups with a diagnosis of inflammatory bowel disease (IBD) or multiple polyps) and individuals with active IBD participate in CRC screening following invitation.

**Materials and methods:**

We used data from the first two years of the Danish CRC screening programme (2014–2015). Information on invitations, participations and FIT test results were obtained from the national screening database, while information on previous CRC, hereditary CRC syndromes, IBD or multiple polyps diagnoses were obtained from the Danish Cancer Registry and the Danish Patient Register. Screening participation rates and FIT-positive rates were calculated and compared for high-risk invitees, invitees having IBD and an average risk group of remaining invitees not diagnosed with colorectal polyps in 10 years preceding the invitation.

**Results:**

When invited to CRC screening, 28–48% of high-risk residents (N: 29; 316; 5584) and 55% of residents with IBD (N: 2217; 6927) chose to participate. The participation rate was significantly higher (67%) among residents without previous colorectal disease, i.e. the average risk group (N = 585,624). In this average group 6.7% of the participants had a positive FIT test. The proportion of positive FIT results was higher among all disease groups (7.7–14.8%), though not statistically significant for participants with prior CRC diagnosis and participants with high-risk IBD.

**Conclusion:**

When high-risk residents and residents with IBD receive an invitation to CRC screening, many participate despite being recommended not to. The screening program was not intended for these groups and further research is needed as several of these groups have a higher rate of positive screening result than the average risk population.

## Introduction

European guidelines, as well as the majority of colorectal cancer (CRC) screening guidelines, recommend CRC screening of average-risk adults only [1, 2]. The European guidelines recommend that 'high-risk individuals should be referred for alternative and more intensive protocols' [1]. High-risk individuals include individuals with a previous diagnosis of CRC or polyps associated with hereditary CRC syndromes as well as certain patient groups previously diagnosed with inflammatory bowel disease (IBD) or multiple polyps [1, 3–7]. As a quantitative faecal immunochemical test (FIT) has been proven an indicator of active IBD [8, 9], participation in FIT-based CRC screening among individuals with active IBD might result in a high number of false-positive cases.

In Denmark, all residents aged 50–74 years are invited to FIT-based CRC screening every second year regardless of risk of CRC [10]. To avoid participation of high-risk individuals with a previous diagnosis of CRC or polyps already enrolled in a surveillance programme, the invitation letter states that the invitation is not relevant for you if you have had CRC or are in a surveillance program due to previous colorectal polyps. Furthermore, the invitation letter states, that if you have Crohns Disease or ulcerative colitis you should discuss with the physician in charge of the surveillance, whether a FIT is relevant for you. However, it is unknown how many high-risk individuals enrolled in a surveillance programme after a CRC or a polyp diagnosis who participate in the screening programme in spite of being informed not to. The proportion of individuals with active IBD that participate in the screening programme is also unknown. In fact, these groups are often excluded from studies investigating the effectiveness of CRC screening programs [11–13]. This leaves their participation rate as well as their gain from the general screening program unknown.

The aim of this article is to investigate the participation rate and subsequent needs for assessments in the Danish national CRC screening programme among high-risk invitees highly likely to be enrolled in a surveillance programme as well as invitees with IBD. The goal is to uncover any discrepancy between recommendations and actual practice and to bring valuable information to other population based screening programmes.

## Materials and methods

### Setting

The Danish FIT-based CRC screening programme was implemented gradually from March 2014 to December 2017. In this period, residents were invited for their first screening in an order based on birth month; all residents born in one month were invited first, then residents born in another month etc. Exempt from this were residents who turned 50 or 75 years during the first years of the screening program. They were invited just before their birthday, in order to secure that the first birth cohorts could follow the screening program as planned and that the oldest cohorts would be screened before they would exit the program [10].

Residents eligible for screening receive an invitation letter by mail from their region of residence together with a four page information leaflet, an OC-sensor FIT sample bottle (Eiken Chemical Company, Tokyo, Japan) and a faeces collection sheet [10]. The samples are then returned to the regional laboratories in charge of analyses. A reminder is sent to invitees who have not returned a sample within 45 days. The FIT is considered positive if the returned sample contains ≥ 20 µg haemoglobin/g faeces. Participants with a positive FIT will be offered a follow-up colonoscopy. Both FIT and any subsequent follow-up colonoscopy and cancer treatment is free of charge due to the tax-payer funded health care system in Denmark [14].

### Study design and population

In this nationwide retrospective cohort study, we analysed to which extent invitees, highly likely to be enrolled in a surveillance programme and invitees with IBD, participated in CRC screening. Among participants, we calculated the proportion having a positive FIT-test. We included all residents born between 1941 and 1963 who were invited to the first round of CRC screening between 10th of March 2014 (start of the screening programme) and 31st of December 2015. As we had data on FIT screening samples up until July 2016, this allowed for a minimum of 6 months of follow-up for all invitees. We did not include invitees who turned 50 or 75 years in 2014 or 2015, as all residents in these birth cohorts were invited within the study period leaving these birth cohorts overrepresented. Also, invitees who died or emigrated within 6 months from the invitation date were excluded.

Included invitees were followed from initial invitation until participation or 6 months after invitation, whichever came first. The study population was grouped into: (1) invitees with a high CRC risk highly likely to be enrolled in a surveillance programme, (2) invitees not belonging to (1) but having an IBD diagnosis prior to the invitation, and (3) invitees not belonging to (1) or (2) and not diagnosed with colorectal polyps in 10 years preceding the invitation (average risk group). Invitees not belonging to (1) or (2) who had colorectal polyps in the 10 years preceding screening invitation were excluded in order to ensure that the 'average risk' group did not include invitees under colonoscopy surveillance. The invitees with colorectal polyps within the last 10 years were excluded as there seems to have been varying local guidelines for surveillance in this group prior to the implementation of the national guideline in 2014.

High-risk invitees highly likely to be enrolled in a surveillance programme (group 1) [3–7] were further categorized into three subgroups based on diagnoses prior to the invitation: (1a) CRC, (1b) ulcerative colitis or Crohn's disease with primary sclerosing cholangitis (PSC) diagnosed within 20 years prior to the invitation [15], or (1c) multiple polyps (> 5) and polyps associated with hereditary CRC syndromes diagnosed within 20 years prior to the invitation [16]. The remaining residents with IBD (group 2) were further categorized into: (2a) residents with a previous diagnosis of ulcerative colitis without PSC and (2b) residents with a previous diagnosis of Crohn's disease without PSC. Table 1 shows the codes defining each group. Colorectal cancer diagnoses were obtained from the Danish Cancer Register while all other diagnoses were obtained from the National Patient Register. Whenever we were interested in older diagnosis we included both ICD7 codes (used in the Cancer Register) and ICD8 codes (used in the Danish National Patient Register) along with the presently used ICD10 codes in Denmark (Table 1).

**Table 1:** Disease groups and defining International Classification of Diseases (ICD) codes

		ICD codes
*Included disease groups*		
1a	CRC	ICD-10: C18*, C19*, C20* ICD-7: 153, 153.0, 153.4, 153.5, 154.9, 253.0, 253.1, 253.2, 253.3, 253.4, 453.0, 453.1, 453.2, 453.3, 453.4, 453.5, 453.8, 454.9, 853.0, 853.1, 853.2, 853.3, 853.4, 853.5, 854.9, 154, 154.0, 454.0, 854.0
1b	IBD with PSC	ICD-10: K50* or K51* combined with DK839F
1c	Multiple polyps and polyps associated with hereditary CRC syndromes	ICD-10 DD126A** (Adenomatosis coli) DD126B** (Polyposis hereditaria coli) DD126C** (Multiple benign neoplasms in the colorectum) DD126F** (Familial adenomatous polyposis)
2a	Ulcerative colitis without PSC	ICD-10: K51* ICD-8: 563.19, 569.04
2b	Crohn's disease without PSC	ICD-10: K50* ICD-8: 563.00–563.02, 563.08, 563.09
*Excluded disease groups*		
Other colorectal polyps within the last 10 years		ICD-10: D120, D121, D122, D123, D124, D125, DD126, D128, D129

Groups rated according to inclusion hierarchy from 1a to 2b. Residents with more than one diagnose are only included in the highest rated disease group

CRC, colorectal cancer; IBD, inflammatory bowel disease (ulcerative colitis or Crohn's diseases); PSC, primary sclerosing cholangitis including all codes starting with the specific letters and numbers

*Including all underlying codes, **DD indicating it is the Danish version of ICD-10, were can include underlying codes annotated with letters in the end

### Definition of outcome

Residents were defined as participants if they returned a sample within 6 months from the invitation date. A participant was considered FIT-positive if the sample contained ≥ 20 µg haemoglobin/g faeces. Residents who only returned an unanalysable sample within 6 months were considered participants, but were not included when calculating FIT-positive rates. FIT-positive rates were therefore calculated as the number of FIT-positive participants divided by the number of residents who returned an analysable sample.

### Data

Data were retrieved from the Danish Colorectal Cancer Screening Database (DCCSD) which is used for monitoring the national CRC screening programme [17]. The data in the DCCSD are gathered from several Danish registers and merged via the unique personal identification number issued to all residents in Denmark. Information on the date of invitation was used to define the study population. These data, as well as information on date and result of any returned screening sample, originated from the IAM (The screening programmes Invitation and Administration Module) database. Information on sex, date of birth, death or emigration originated from the Danish Civil Registration System [18]. Information on adenomas and IBD prior to invitation was obtained from the Danish Patient Register [19] and information on previous CRC diagnosis from the Danish Cancer Registry [20].

### Availability of data and materials

The data that support the findings of this study are available from the DCCSD [21] and The Danish Health Data Authority [22]. Restrictions apply to the availability of these data, which were used under license for this study. Data may be available upon reasonable request to the DCCSD and The Danish Health Data Authority.

### Statistics

We calculated 95% Clopper-Pearson binomial confidence intervals for participation rates as well as FIT-positive rates. Multiple logistic regression adjusting for age and gender was used to calculate odds ratio for participation and for having a FIT positive test among high-risk and IBD groups compared to the average risk group.

### Ethics

According to EU's General Data Protection Regulation (article 30), the project was listed at the record of processing activities for research projects in Central Denmark Region (J. No.: 1-16-02-396-16). According to the Danish Consolidation Act on Research Ethics Review of Health Research Projects, Consolidation Act number 1083 of 15 September 2017 section 14 (2) notification of questionnaire surveys or medical database research projects to the research ethics committee system is only required if the project involves human biological material. Therefore, this study may be conducted without an approval from the committees. As this is an entirely register-based study, informed consent from participants is not required in Denmark. Data were pseudonymized and placed on a secured server at the Danish Health Data Authorities. When retrieved from the secured server, data were anonymized. All methods were performed in accordance with the relevant guidelines and regulations.

## Results

618,578 Danish residents born between 1941 and 1963 were invited for CRC screening between 10th of March 2014 and 31th of December 2015. Of these, 3,680 (0.6%) were excluded due to death or emigration in the follow-up period and 14,202 (2.3%) were excluded due to a colorectal polyp diagnosis within the last 10 years as the only known colorectal disease. In total, 600,696 were included in the analysis. 585,624 of these were included in the 'average risk' group, while 15,072 were grouped according to history of colorectal disease. Prior to invitation, 5583 (37.0%) of these had a previous CRC diagnosis; 29 (0.2%) had IBD with PSC; 316 (2.1%) had multiple polyps and polyps associated with hereditary CRC; 6927 (46.0%) had ulcerative colitis; and 2217 (14.7%) had Crohn's disease (Table 2). Mean age was higher among invitees with previous CRC and 'multiple polyps and polyps associated with hereditary CRC' than in the average risk group (Table 2). The proportion of men was highest in the various high-risk groups while it was lowest in the IBD groups (Table 2).

**Table 2:** Mean age with standard deviation (SD) and proportion of women and men

Groups		Invited	Age		Proportion of men
		N	Mean	SD	%
3	Average risk	585,624	62.01	6.63	49.1
1a	CRC	5583	66.26	5.80	54.4
1b	IBD with PSC	29	60.31	5.99	58.6
1c	Multiple polyps and polyps associated with hereditary CRC	316	64.80	6.45	56.3
2a	Ulcerative colitis without PSC	6927	61.81	6.51	46.3
2b	Crohn's disease without PSC	2217	61.31	6.54	44.2

CRC, colorectal cancer; IBD, inflammatory bowel disease (ulcerative colitis or Crohn's disease); PSC, primary sclerosing cholangitis

In total 67% of the invitees in the 'average risk' group returned a sample within 6 months (Table 3). This was significantly higher than in any of the disease groups. Among invitees with ulcerative colitis and Crohn's disease 55% participated. This was significantly lower than the participation rate in the 'average risk' group even when adjusting for age and gender (OR_adjusted_: 0.597 (95% CI 0.569; 0.627) and OR_adjusted_: 0.594(95% CI 0.546; 0.647)). Invitees with multiple polyps/polyps associated with hereditary CRC the participation rate was 48%. This was significantly higher than the participation rate among invitees with prior CRC diagnosis( 29%). Invitees with IBD and PSC had a participation rate of 31% (N: 29) (Table 3).

**Table 3:** Participation among residents invited for colorectal cancer screening

Groups		Invited	Participants		Adjusted* OR
		*N*	*n*	*% (95% CI)*	
3	Average risk	585,624	391,514	66.9 (66.7; 67.0)	1
1a	CRC	5583	1594	28.6 (27.4; 29.8)	0.184 [0.173; 0.195]
1b	IBD with PSC	29	9	31.0 (15.3; 50.8)	0.228 [0.103; 0.503]
1c	Multiple polyps and polyps associated with hereditary CRC	316	151	47.8 (42.2; 53.4)	0.437 [0.350; 0.546]
2a	Ulcerative colitis without PSC	6927	3808	55.0 (53.8; 56.1)	0.597 [0.569; 0.627]
2b	Crohn's disease without PSC	2217	1213	54.7 (52.6; 56.8)	0.594 [0.546; 0.647]

CRC, colorectal cancer; IBD, inflammatory bowel disease (ulcerative colitis or Crohn's disease); PSC, primary sclerosing cholangitis

*Adjusted for sex and 5-year age group

In the 'average risk' group, 6.7% of the participants who returned an analysable stool sample had a positive screening test (Table 4). Of the participants with a prior CRC diagnosis who returned an analysable stool sample, 7.7% had a positive screening result. The odds for a FIT-positive result was not statistically significant from the average risk group when adjusting for age and gender, OR_adjusted_ 0.9 (95% CI 0.8; 1.1). Participants with multiple polyps/hereditary polyp syndromes, ulcerative colitis and Crohn's disease had a statistically significant higher proportion of FIT-positive tests compared to the 'average risk' group with a positivity rate of 13.2%, 14.8% and 12.9% and OR_adjusted_ at 2.0, 2.5 and 2.2, respectively (Table 4). The small group of residents with IBD and PSC had a highly increased positivity rate but the numbers were too small to report and the 95% CI was wide. As a result, the estimate was not statistically significantly different from the 'average risk' group.

**Table 4:** Proportion of positive colorectal cancer screening tests

Groups		Participants	FIT positive		Adjusted* OR
		*N*	*n*	*% (95% CI)*	
3)	Average risk	391,514	26,379	6.7 (6.7; 6.8)	1
1a)	CRC	1594	122	7.7 (6.4; 9.1)	0.949 [0.789; 1.142]
1b)	IBD with PSC	9	< 5**	NA	4.015 [0.826; 19.515]
1c)	Multiple polyps and polyps associated with hereditary CRC	151	20	13.2 (8.3; 19.7)	1.962 [1.234; 3.120]
2a)	Ulcerative colitis without PSC^7^	3808	562	14.8 (13.6; 15.9)	2.482 [2.267; 2.717]
2b)	Crohn's disease without PSC	1213	157	12.9 (11.1; 15.0)	2.181 [1.843; 2.581]

FIT, faecal immunochemical test; CRC, colorectal cancer; IBD, inflammatory bowel disease (ulcerative colitis or Crohn's disease); PSC, primary sclerosing cholangitis

*Adjusted for sex and 5-year age group, **Exact number not reported due to small number

## Discussion

### Main findings

Almost one third of the residents with a previous CRC diagnosis participated in CRC screening even though this high-risk group is not in the target group for CRC screening and are specifically told not to participate in the invitation letter. About half of the invitees with multiple polyps/polyps associated with hereditary CRC participated in CRC screening although most of them are recommended to follow other surveillance programmes [16]. Among invitees with ulcerative colitis and Crohn's disease, approximately 50% participated in CRC screening. The FIT-positive rate was approximately doubled among participants with ulcerative colitis, Crohn's disease and among residents with multiple polyps/hereditary polyp syndromes compared to the average risk group.

### Strengths and limitations

The major strength of this study is the inclusion of all invitees in 2014 and 2015 and the use of high-quality registers [18–20] for identification of previous colorectal disease. Due to this, we avoided selection bias and recall bias.

We did not know the disease activity of the included invitees with IBD as this information is not included in the registers. Residents having a low disease activity may have been recommended to participate in CRC screening. Given the high FIT-positive rate among participants with IBD, invitees with high disease activity are likely to have participated in the CRC screening. This might be problematic as FIT has been shown to correlate with clinical status of IBD in the colon [8, 23].

Residents with Crohn's disease and PSC as well as residents with ulcerative colitis and PSC are recommended annual colonoscopy surveillance and are therefore not recommended to participate in CRC screening [15]. Despite this, 9 out of 29 in this group chose to participate in CRC screening. Unfortunately, the absolute numbers in this group regarding FIT results was too small to report and further studies regarding this high risk group would require a longer inclusion period.

As we only had information on participation until July 2016, we defined participation as participation within 6 months from the date of invitation. If including only those invited at least 12 months prior to July 2016 and allowing for participation up to 12 months after the date of invitation, participation rates were 1–2% higher. However, the difference within the groups remained the same (data not shown).

Participation rates could be different in subgroups within the disease groups depending on socioeconomic status and comorbidities. However, the numbers in our subgroups were too small to look into this.

### Interpretations and implications

Despite not knowing whether invitees with a previous colorectal disease are following a surveillance programme and whether residents with IBD have a low or high disease activity, it seems that a too large proportion of these residents chose to participate in CRC screening.

In Denmark, resident with multiple polyps/polyps associated with hereditary CRC are most likely recommended for some kind of colorectal surveillance [16]. Therefore, a participation rate of nearly half this population indicates, that many of them does not comply with the recommendations.

The study raises several questions regarding the incentive for participation for the invitees. Is the participation in the screening program a supplement or a substitute for the disease specific surveillance? Do they participate with the endorsement from the healthcare professionals, or is it easier to take the test, than contacting the general practitioner for advice? If the test is positive, how are they handled in the screening setting not designed for their initial disease?

If the easy and convenient FIT-screening is taken as a substitute to a surveillance colonoscopy, the test could possibly do more harm than good for the high risk participants, as the FIT screening has been proven to be less sensitive in a post polypectomy surveillance population than surveillance colonoscopy [24]. This needs to be communicated in light of the result from the present study.

Even though subgroups of IBD patients have an elevated risk of CRC, especially among patients with ulcerative colitis [25, 26], patients with Crohn's disease have been shown to have a CRC risk comparable to that of the general population [25]. Therefore, a significantly increased FIT-positive rate may indicate that IBD patients will have a higher false-positive rate as well. This is in line with the German BliTz study that recently showed that unknown IBD disease was an independent risk factor for apparent false-positive results in a FIT-based CRC screening programme. However, the result was based on only eight cases of unknown IBD [27]. It is a topic for further research to study whether IBD patients have a higher false-positive rate than average-risk residents without these diseases. It is essential factors to take into account, when communicating to the invitees as well as health care professionals advising their patients on whether or not to participate in the population based CRC screening program.

When residents who are recommended not to participate in a population based CRC screening still choose to participate, it could constitute an unnecessary burden for both society and residents, if they are still following the disease specific surveillance. If they do in fact not follow the recommended disease specific surveillance, the participation in the CRC screening program could instead lead to insufficient surveillance.

To our knowledge, this is the first study investigating the participation and FIT screening results among invitees whom are not in the target group of a population based CRC screening. It is a unique issue within CRC screening, as other cancer screening tests, so far, has been performed by health care professionals. But with home-send and at home-performed FIT screening, the invitation letter is suddenly the primary gatekeeper when it comes to ensuring invitees out of scope for the screening program do not participate. With the implementation of self-sampling HPV screening, this could however, become an issue within cervical cancer screening as well.

## Conclusion

When invited, many residents with a high risk of CRC chose to participate in CRC screening despite not being in the target group for CRC screening and despite being recommended not to participate in the invitation letter. The FIT positive rate was higher in all disease groups than in the average risk group, though the difference was not statistically significant for the groups with prior CRC and IBD with PSC. These high FIT positive rates are either due to high false positive rates or true positive rates in the disease groups. Further studies are warranted to tell whether these high participation rates results in a high false positive rates or high true positive rates.

## Data Availability

The data that support the findings of this study are available from The Danish Colorectal Cancer Screening Database (DCCSD) and The Danish Health Data Authority. Restrictions apply to the availability of these data, which were used under license for the current study, and so are not publicly available. Data may be available upon reasonable request to The DDCSD Dansk Tarmkræftscreeningsdatabase (DTS)—RKKP and Danish Health Data Authority English—Sundhedsdatastyrelsen.

## Abbreviations

CRC: Colorectal cancer
IBD: Inflammatory bowel disease
PSC: Primary sclerosing cholangitis
DCCSD: Danish Colorectal Cancer Screening Database
IAM: Invitation and Administration Module
